# Reorganisation of Wnt-response pathways in colorectal tumorigenesis

**DOI:** 10.1038/sj.bjc.6604327

**Published:** 2008-04-15

**Authors:** G M Caldwell, C E Jones, Y Soon, R Warrack, D G Morton, G M Matthews

**Affiliations:** 1 Division of Medical Sciences, Epithelial Research Group, School of Medicine, The University of Birmingham, Edgbaston, Birmingham B15 2TH, UK

**Keywords:** colorectal carcinogenesis, Wnt signalling, NKD1, *β*-catenin-independent signalling

## Abstract

In most colorectal tumours, APC mutation stabilises *β*-catenin and mimics elements of Wnt growth factor signalling, but the high frequency of epigenetic loss of Wnt antagonists indicates an additional role for ligand-mediated Wnt signalling. Here, we have investigated the expression of key components of *β*-catenin-independent Wnt response pathways to determine whether their profiles change during the transition from normal mucosa to colorectal adenomas. Transcription of the Wnt/planar cell polarity pathway determinant NKD1 (naked cuticle homologue 1) was induced in adenomas by a median 135-fold and in cancers by 7.4-fold. While some Frizzleds (FZDs) were downregulated in adenomas, the Wnt/Ca^2+^ receptors FZD3 and FZD6 were induced by a median factor of 6.5 and 4.6, respectively. Naked cuticle homologue 1, FZD3 and FZD6 expression were coordinated in pre-malignant disease, but this relationship was lost in invasive cancers, where FZD induction was seen less frequently. Naked cuticle homologue 1 expression was associated with nuclear localisation of phospho-c-Jun in adenomas. In cultured cells, NKD1 transcription was induced by lithium chloride but FZD3 expression required Wnt growth factor treatment. These data show that Wnt responses are consistently directed towards both *β*-catenin-independent routes in early colorectal tumorigenesis and elements of this are retained in more advanced cancers. These *β*-catenin-independent Wnt signalling pathways may provide novel targets for chemoprevention of early colorectal tumours.

Our studies (Caldwell *et al*, 2004, 2006) and those of others (Suzuki *et al*, 2004) have established that Wnt antagonists are downregulated at the earliest stages of colorectal tumorigenesis, presenting the neoplastic cells with an environment where Wnt growth factor signals are unmodulated. These data indicate a potential role for Wnt growth factor signalling in tumorigenesis and this is supported by the sensitivity of tumour cells to exogenous antagonists of Wnt—Frizzled (FZD) interaction (DeAlmeida *et al*, 2007).

Wnt growth factors have been shown to influence *β*-catenin activity, when normal controls have been lost through *β*-catenin mutation as occurs sometimes in colorectal cancer (Suzuki *et al*, 2004), but do not appear to influence this pathway in the presence of APC mutations (DeAlmeida *et al*, 2007), which are more common in genetic and sporadic disease. Wnts can, however, also provoke responses through alternative, *β*-catenin-independent, signalling pathways. Of these, the Wnt/planar cell polarity (Wnt/PCP) pathway (Boutros *et al*, 1998) employs the same FZD receptors as the *β*-catenin pathway, the signal being diverted within the cell by the action of naked cuticle homologue 1 (NKD1) (Yan *et al*, 2001), resulting in activation of Jun N-terminal kinase (JNK) and RhoA, while the Wnt/calcium (Wnt/Ca^2+^) pathway is more difficult to define, particularly in non-embryonic tissues. It may employ a distinct subset of FZDs, notably FZD3, FZD4 and FZD6 (Sheldahl *et al*, 1999), and can lead to a rise in cytosolic calcium levels (Sheldahl *et al*, 1999) and/or antagonism of *β*-catenin action (Mikels and Nusse, 2006).

The central role of *β*-catenin driven transcription in colorectal tumorigenesis has been firmly established, but the contributions of the alternative Wnt pathways have not been explored. Recently, c-Jun has been shown to be important in bowel adenoma formation and to cooperate with *β*-catenin in promoting TCF4-dependent transcription (Nateri *et al*, 2005; Toualbi *et al*, 2007), implying a role for the Wnt/PCP pathway, although the potential for Wnt/Ca^2+^ signalling to participate is less clear. One of its known outcomes is suppression of *β*-catenin action (Mikels and Nusse, 2006), but it is possible that chronic activation of this pathway might have other effects in neoplasia. In non-small cell lung cancer, expression of one of the Wnt/Ca^2+^ pathway's ligands, Wnt5a, has been associated with disease progression (Huang *et al*, 2005). This has also been reported in gastric cancer (Kurayoshi *et al*, 2006) and pancreatic cancer (Ripka *et al*, 2007). It may, however, act as a tumour suppressor in other cases (Liang *et al*, 2003; Blanc *et al*, 2005; Dejmek *et al*, 2005; Kremenevskaja *et al*, 2005), suggesting that Wnt/Ca^2+^ signalling can have a variable role in different tumour settings.

In normal cells, both the Wnt/PCP and Wnt/Ca^2+^ pathways participate in homoeostatic responses to Wnt/*β*-catenin signalling, by diverting the signal downstream of the receptor and by antagonising *β*-catenin/TCF4 transcription. We hypothesised that the chronic state of *β*-catenin activation in colorectal tumour cells might, therefore, lead to a sustained increase in expression of these alternative Wnt-response pathways, which, in the absence of secreted antagonists such as secreted Frizzled-related protein 1 (sFRP1) (Caldwell *et al*, 2004, 2006), would be highly sensitive to Wnt growth factor signalling and this may contribute to tumorigenesis. Naked cuticle homologue 1 is central to the Wnt/PCP pathway and has previously been shown to be expressed in advanced colorectal cancers (Yan *et al*, 2001) and to be induced by the Wnt/*β*-catenin pathway (Caldwell *et al*, 2006), but its expression in premalignant adenomas has not been investigated. Similarly, expression changes in the FZDs, particularly FZDs 3, 4 and 6 as determinants of a Wnt/Ca^2+^ response, have not been measured in colorectal adenomas or cancers.

In this paper, we show that NKD1, FZD3 and FZD6 transcripts are all induced significantly in colorectal adenomas compared to matched normal tissue, indicating that both the Wnt/PCP and the Wnt/Ca^2+^-response pathways are expressed at an early stage in tumour formation. These inductions are coordinated in adenomas and correlate with *β*-catenin stabilisation and nuclear accumulation of phospho-c-Jun. Stabilisation of *β*-catenin in cultured cells using lithium chloride induced NKD1 (Wnt/PCP pathway) expression but had no effect on FZD3 (Wnt/Ca^2+^ pathway) levels, while Wnt3a conditioned medium caused induction of both targets, indicating that *β*-catenin is responsible for some but not all of the changes seen in adenomas.

These data show that *β*-catenin stabilisation is sufficient to induce Wnt/PCP components but that a Wnt signal is required to induce expression of the Wnt/Ca^2+^ receptors, indicating that the expression profile of Wnt/Ca^2+^ receptors seen in adenomas requires *β*-catenin-independent signalling.

## MATERIALS AND METHODS

### Sample collection

Adenoma and carcinoma samples were obtained with individual patient consent, following the local ethics committee guidelines for resected specimens. Separate ethical approval was given for analysis of paraffin blocks (LREC No. 2003/277).

All adenomas were from the rectum and sigmoid colon. The 14 matched samples were from patients with a median age 63 years (range 56–65 years), and included five adenomas less than 10 mm in diameter, five tubular adenomas and nine tubulovillous lesions. Unmatched polyps included six adenomas under 10 mm in size, two were simple tubular lesions and the median age was 61 years. The unmatched normal tissue was from patients with a median age of 63 years (range 60–65 years).

Eighteen primary colorectal cancers were collected with matched normal mucosal samples. The median age of the cohort was 74 years (range 43–91 years). In 10 cases, the primary cancer was at the splenic flexure or proximal colon, and 3 were rectal cancers. Six cancers had lymph node metastases at the time of diagnosis, and two had liver metastases.

### RNA extraction and reverse transcription

Total RNA was extracted using TRI reagent (Sigma, Poole, UK) according to the manufacturer's instructions. First-strand cDNA was synthesised from 2 *μ*g of DNase-treated total RNA using Ready-To-Go You-Prime First-Strand Beads (Amersham Pharmacia Biotech, Chalfont, St Giles, UK) and random hexamers (Promega, Southampton, UK).

### Qualitative RT–PCR

Oligonucleotide primers to amplify NKD1 and FZDs 3, 4, 6 and 10 were designed using Primer3 software (Rozen and Skaletsky, 1996) and synthesised by Alta Biosciences (University of Birmingham, Birmingham, UK). Details of these are given in Table 1. Where possible, amplicons were designed to span an exon/intron boundary. Oligonucleotides for FZDs1, 2 and 8 were as previously published (Kirikoshi *et al*, 2001). All reactions were prepared to a final volume of 25 *μ*l, containing 1 × BIOTAQ NH4-based reaction buffer (BIOLINE, London, UK), 1 mM Mg_2_Cl, 2 mM dNTPs (Amersham Pharmacia Biotech), 250 ng of each primer, 0.5 U of BIOTAQ DNA polymerase (BIOLINE) and 1 *μ*l of cDNA. Amplification involved an initial step of 2 min at 94°C, followed by 35 cycles of 94°C for 10 s, 58°C for 20 s and 72°C for 30 s, with a final extension of 5 min at 72°C.

### Quantitative real-time PCR

Oligonucleotide primers and TaqMan probes (Table 1) were designed using Primer Express™, version 1.5 (PE Applied Biosystems, Warrington, UK). TaqMan Universal PCR Master Mix and TaqMan probes were purchased from PE Applied Biosystems. Multiplex PCR amplifications were performed using an ABI PRISM 7700 Sequence Detector in a final volume of 25 *μ*l. Each reaction contained 12.5 *μ*l of 2 × TaqMan Universal PCR Master Mix (PE Applied Biosystems), 90 nM keratin 8 (KRT8) and target gene primers, 150 nM target gene TaqMan probe, 175 nM KRT8 TaqMan probe and 1 *μ*l of cDNA. Cycling conditions were an initial step at 50°C for 2 min and 95°C for 10 min, followed by 40 cycles at 95°C for15 s and 60°C for 1 min. Results from the target genes were normalised to the epithelial cell-specific gene KRT8 as described previously (Caldwell *et al*, 2004). The expression levels of *KRT8* were consistent between adenoma (median *C*_t_ value=22.40, IQR 20.3–23.2) and normal samples (median *C*_t_ value=22.39, IQR 21.8–23.8).

### Immunohistochemistry

The Streptavidin–biotin indirect immunoperoxidase method was performed as described previously (Hardy *et al*, 2002). Sections (5 *μ*m) were dewaxed, rehydrated and endogenous peroxidase activity blocked by incubation in 10% H_2_O_2_ in methanol for 10 min. Microwave antigen retrieval was undertaken for 1 h. Sections were incubated overnight with primary antibodies recognising *β*-catenin (clone 14, Transduction Laboratories, Cowley, UK) or phospho-c-Jun (ser63-P, Santa Cruz Biotechnology, Heidelberg, Germany) at a dilution of 1 : 300. After washing with PBS, sections were incubated with biotinylated goat anti-mouse/rabbit IgG (Dako, Ely, UK) according to the manufacturer's instructions for 30 min. Serial PBS washing and incubation with streptavidin-peroxidase conjugate (Dako) was undertaken prior to incubation with diaminobenzidine tetrahydrochloride (Sigma). Sections were counterstained with haematoxylin (BDH), dehydrated and analysed on a light microscope.

### Lithium and Wnt3a treatment of cultured cells

The normal small intestine epithelial cell line FHs74Int (ATCC, LGC Promochem, Teddington, UK) was cultured using Hybri-Care medium (ATCC) supplemented with 1.5 g l^−1^ sodium bicarbonate and 10% foetal calf serum. Cells were grown in 6 cm dishes and treated with 10 mM LiCl or 10 mM KCl as a control. Cells were harvested over a time course of 16 h and RNA, DNA and protein were extracted using TRI reagent (Sigma).

Wnt3a conditioned medium was produced from mouse L-cells (ATCC) stably expressing the recombinant protein and used to treat HeLa cells. Parallel experiments were performed with medium conditioned by the parental L-cells to exclude the effects of other secreted activities.

Where appropriate, statistical analysis was performed using Fishers exact test.

## RESULTS

### RT–PCR analysis of colorectal adenomas

**Figure 1:**
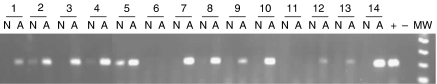
RT–PCR detection of NKD1 in colorectal adenomas. cDNA prepared from 14 matched normal/adenoma pairs (N and A, respectively) was amplified using primers specific for NKD1 together with a positive control (+) and a sample with no cDNA (−) and analysed by agarose gel electrophoresis. Products can be observed in all adenoma tracks except for samples 6 and 11 but are absent from the normal samples except for 2, 4 and 5.

We devised conventional RT–PCR assays for NKD1 and each of the human FZD sequences and used these to analyse RNA extracted from a series of adenomas and control normal mucosa samples. Figure 1 shows the results of this analysis for NKD1 on a series of 14 adenomas. Of these, the majority (12 out of 14) of adenomas expressed NKD1, but this was detected in only three of the matched normal controls (*P*=0.001).

Significant changes were also apparent in the analyses performed on a series of 19 adenomas for the FZDs, and these are summarised in Table 2 (primary data not shown). While the frequency of FZDs 1, 5 and 7 expression appeared unchanged between normal and adenoma RNA, FZDs 2 and 8 were expressed much less often in the adenomas (*P*=0.003 and 0.007 respectively). Frizzleds 4, 6 and 10 showed less obvious changes, with a tendency to become expressed in the adenomas, the increased incidence of FZD6 just reaching significance (*P*<0.04). The most striking change found, however, was that for the Wnt/Ca^2+^ receptor FZD3, which was expressed in 13 of 19 adenomas, but only in 2 of 13 normal tissues (*P*=0.005).

### Real-time RT–PCR analysis

**Figure 2:**
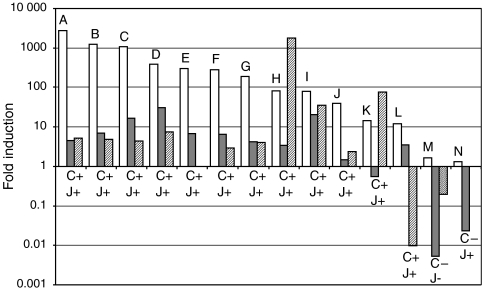
Real-time RT–PCR analysis of NKD1, FZD3 and FZD6 expression in colorectal adenomas. Relative expression of NKD1 (open bars), FZD3 (grey bars) and FZD6 (striped bars) was determined in a series of adenomas by comparison to their levels in matched normal samples, using real-time RT–PCR as described in the text. Samples were ordered by NKD1 expression levels to reveal any association between the targets. Note that two samples (E and N) were not analysed for FZD6. These adenomas were also stained to assess nuclear accumulation of *β*-catenin and phospho-c-Jun and the outcome of this is indicated below each cluster of bars where + indicates detection and − indicates a failure to detect nuclear *β*-catenin (C) and phospho-c-Jun (J).

Since the qualitative review of expression indicated induction of NKD1, FZD3 and FZD6 in colorectal adenomas, we developed quantitative real-time RT–PCR assays for each of these to determine the extent of the induction. Transcript levels were compared with levels of KRT8, an epithelial cell-specific marker, to control for the variation in stromal and inflammatory cell components between dysplastic and normal epithelium (Caldwell *et al*, 2004, 2006). Performing these experiments for NKD1 revealed substantial induction in all but 2 of 14 adenomas, compared with normal mucosa. In two-thirds (9 out of 14) of cases, NKD1 was upregulated by a factor of more than 50-fold, and in half (7 out of 14), the induction was in excess of 100-fold, while the median level of induction over the whole group was 135-fold (Figure 2).

Frizzleds 3 and 6 were also induced in most adenomas, with a median of 6.5- and 4.6-fold, respectively. Frizzled 3 was induced in 11 of 14 adenomas by a factor of 1.5–31. Frizzled 6 was measured in a subset of 12 of the 14 adenomas and was found to be induced in 10 of these.

These data show upregulation of key components of both the Wnt/PCP and the Wnt/Ca^2+^ pathways compared to normal epithelium.

### *β*-Catenin stabilisation

Although most colorectal tumours harbour APC mutations, some develop through alternative routes (Morin *et al*, 1997). We stained paraffin sections of the series of 14 adenomas analysed previously to identify nuclear *β*-catenin and identified this in all but two cases (M and N). These were the same individuals that lacked induction of NKD1, FZD3 and FZD6 mRNAs, indicating that unregulated *β*-catenin signalling is required to induce these transcripts.

### Signalling in adenomas

**Figure 3:**
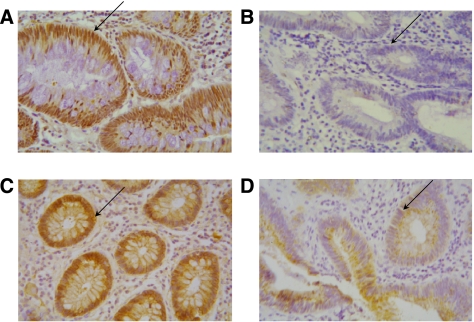
Immunohistochemical detection of *β*-catenin and phospho-c-Jun in colorectal adenomas. Paraffin sections of fixed adenoma tissue were stained to detect phospho-c-Jun (**A** and **B**) and *β*-catenin (**C** and **D**). Representative samples are shown. Positive nuclear localisation was detected in sample (**B**), shown by the arrow (**A** and **C**), whereas in sample M (**B** and **D**) the arrow indicates the absence of nuclear accumulation.

Signalling outcomes of *β*-catenin-independent Wnt pathways in human epithelia have yet to be defined. The Wnt/Ca^2+^ pathway can lead to a range of outcomes, some of which, such as changes in cytosolic calcium levels, cannot be measured in adenoma biopsies. Wnt/PCP signalling, however, invariably involves JNK activation. We investigated the presence of nuclear phosphorylated c-Jun, as a marker of this activity, in paraffin sections of the adenomas used previously for RNA analysis, and examples of this are shown in Figure 3. All but one of the adenomas contained nuclear phospho-c-Jun. This exception was one of the two adenomas where *β*-catenin was not seen in the nucleus, which also contained low levels of NKD1, these data are summarised in Figure 2.

### Induction of NKD1 and FZD3 in cultured cells

**Figure 4:**
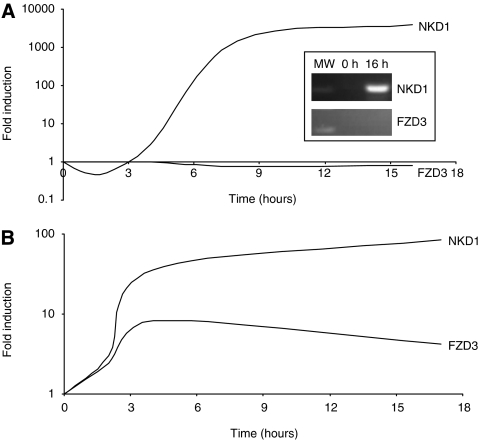
Induction of NKD1 and FZD3 in cultured cells. (**A**) Lithium chloride induction of NKD1 mRNA in FHs74Int cells. Confluent cells were treated with lithium chloride and sampled over a 16-h time course. Expression of FZD3 and NKD1 was measured by real-time RT–PCR and the level relative to untreated control cells determined. The inset panel shows qualitative RT–PCR analysis of the induced cells at 0 and 16 h. (**B**) Wnt3a induction of NKD1 and FZD3 mRNA in HeLa cells. Confluent cells were treated with Wnt3a-conditioned medium and sampled over a 17-h time course. Expression of NKD1 and FZD3 was measured by real-time RT–PCR and the level relative to untreated control cells determined.

The correlation between NKD1, FZD3 and FZD6 and *β*-catenin stabilisation indicates a cell autonomous effect in the tumour epithelium. In the absence of reliable reagents to assess this in histological sections, we attempted to recapitulate these events *in vitro*. Previous experiments (Yan *et al*, 2001) demonstrated *β*-catenin dependence of NKD1 transcription. Stabilisation of *β*-catenin in cultured cells would determine whether the FZD expression changes are cell autonomous. The epithelial cell line Fhs74Int, which was isolated from normal intestine (Owens *et al*, 1976) and retains normal APC function, was used for these experiments. We treated confluent cultures with lithium chloride, an inhibitor of glycogen synthase kinase-3*β* (GSK-3*β*), to stabilise *β*-catenin (Klein and Melton, 1996), and measured changes in NKD1 and FZD3. Figure 4A shows that NKD1 levels increased substantially, reaching a peak after 8 h and dropping gradually towards 16 h after induction, while FZD3 levels were essentially unaffected by lithium treatment, indicating that *β*-catenin stabilisation alone cannot account for the induction of FZD3 observed in adenomas.

In a separate experiment, shown in Figure 4B, HeLa cells, which have intact APC and normal *β*-catenin levels, were treated with medium conditioned by expression of recombinant Wnt3a from mouse L-cells. In this case, NKD1 levels continued to rise over the 17 h time course of the experiment, while FZD3 expression was maximal after 4 h of treatment. In both cases, the levels of induction reached were comparable with those seen in our previous analysis of adenomas.

### Expression in cancers

**Figure 5:**
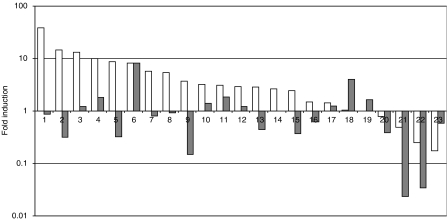
Real-time RT–PCR analysis of NKD1 and FZD3 expression in colorectal cancers. Relative expression of NKD1 (open bars) and FZD3 (filled bars) was determined in a series of colorectal cancers by comparison to matched normal samples, using real-time RT–PCR as described in the text. Samples were ordered by NKD1 levels for comparison between the two series.

To determine whether this induction pattern is sustained throughout tumorigenesis, we performed real-time RT–PCR analysis of NKD1 and FZD3 in a series of 23 colorectal cancers. Figure 5 shows that while NKD1 was induced in the majority (20 out of 23) of cancers compared with matched normal tissue, the median level of induction (7.4-fold) was substantially lower than the 135-fold we saw in adenomas. The pattern of FZD3 induction was also markedly different in cancers, with only 40% (9 out of 23) showing significant induction, 30% (7 out of 23) essentially unchanged and the remaining 30% (7 out of 23) downregulated. In addition, there was no clear relationship between NKD1 and FZD3 induction, contrasting with the expression profiles seen in adenomas.

## DISCUSSION

Our data show that NKD1, FZD3 and FZD6, key determinants of the *β*-catenin-independent Wnt response pathways, are induced early in colorectal tumorigenesis, coinciding with the loss of Wnt antagonists such as sFRP1 (Caldwell *et al*, 2006). As adenomas progress into cancers, NKD1 expression reduces and its relationship with FZDs3 and 6 levels is lost. *In vitro* experiments indicated that while NKD1 induction is a direct result of *β*-catenin signalling, FZD3 expression requires a Wnt growth factor signal. This indicates that Wnt/Ca^2+^ receptor expression in tumours occurs in response to the unmodulated Wnt signal that results from Wnt antagonist downregulation.

A previous study (Yan *et al*, 2001) showed NKD1 expression in colorectal cancer and regulation by *β*-catenin *in vitro*, but our study is the first to examine its expression in adenomas and quantify the degree of induction. The extent of upregulation we found in adenomas (median 135-fold) was striking and indicates that incoming signals from Wnt growth factors would be directed towards the Wnt/PCP response in these tumours. Our demonstration of c-Jun activation in adenomas supports this. The importance of c-Jun in colorectal adenoma formation, through its interaction with *β*-catenin and TCF4, has been demonstrated in animal models (Nateri *et al*, 2005), but Wnt/PCP signalling is not the only route to c-Jun activation and its contribution to this in adenomas warrants further investigation.

The median level of NKD1 induction in cancers (7.4-fold) was substantially lower than the 135-fold we saw in adenomas. The reason for the difference observed between the degree of change seen in adenomas and in cancers is not entirely clear. The many differences between adenomas and cancers may lead to a reduction in dependence on these reorganised Wnt pathways as the disease progresses. Although genetic changes (e.g. APC mutations) cannot be reversed during tumour progression, other effects may show plasticity when selection pressure is lost due to mutations in other pathways. To explore this, we shall first have to define the outcomes of *β*-catenin-independent Wnt signalling in the adenoma.

The potential for Wnt/Ca^2+^ signalling to have a role in early colorectal tumorigenesis is presently unclear. Signalling in this pathway has been associated with the progression in some tumours (Huang *et al*, 2005; Kurayoshi *et al*, 2006; Ripka *et al*, 2007) and suppression in others (Liang *et al*, 2003; Blanc *et al*, 2005; Dejmek *et al*, 2005; Kremenevskaja *et al*, 2005), so any contribution it makes to tumorigenesis may depend on other factors in the tissue.

One disadvantage of studying these events in adenomas is the scarcity of material available, which limits the number of parallel analyses that can be performed on a single sample. For this reason, we have chosen not to explore Wnt/Ca^2+^ signalling outcomes in this cohort until we can identify unambiguous markers of a response. Studies in more tractable systems have shown that Wnt5a and Wnt11 action leads to a loss of cell adhesion, stimulates PKC and CamKII, and antagonises the *β*-catenin pathway (Torres *et al*, 1996). Ca^2+^-dependent PKC isoforms are present in colorectal adenomas, but are less abundant in proliferating areas (Kahl-Rainer *et al*, 1996), so it is unlikely that a simple relationship exists between Wnt/Ca^2+^ signalling through PKC and proliferation of adenoma cells. Studies with purified Wnt5a indicated that calcium release is not a universal outcome of signalling, but demonstrated receptor-dependent antagonism of *β*-catenin's transcriptional activity in the presence of the ROR2 co-receptor (Mikels and Nusse, 2006) emphasising the impact of the receptor context on signalling outcomes. We investigated the expression of ROR2 in our tumours but found no consistent pattern of expression, with as many cases showing downregulation as increased expression (data not shown). Since unregulated *β*-catenin-dependent transcription is a hallmark of colorectal tumours, *β*-catenin antagonism must be incomplete.

In cultured cells, stabilisation of *β*-catenin using lithium treatment led to NKD1 expression but failed to influence Wnt/Ca^2+^ receptors, while Wnt growth factor treatment induced FZD3 transcripts to the degree seen in adenomas. In early tumorigenesis, Wnt signalling is enhanced relative to normal epithelium as a result of antagonist suppression, and this is likely to be the origin of the elevated FZDs3 and 6 expression we identified in adenomas.

In normal cells, NDK1 and FZD3/6 are expressed transiently to repress further *β*-catenin stabilisation providing a negative feedback to Wnt stimulation. In tumour cells lacking APC function, this homoeostatic loop fails to complete, leading to sustained expression and establishment of alternative Wnt-response pathways. The epigenetic suppression of secreted Wnt antagonists seen in adenomas (Caldwell *et al*, 2006) and the sensitivity of tumour cells to Wnt restriction (DeAlmeida *et al*, 2007) indicate an enduring requirement for Wnt ligand signals, which will lead to responses in the pathways induced. This may have a number of consequences, such as stimulation of MAP kinase signalling by mobilising EGF ligands (Kim and Choi, 2007; Schlange *et al*, 2007).

The high levels of *β*-catenin-independent Wnt-response pathway components we have shown distinguish early tumour cells from normal tissue. These early tumours are potentially unstable and susceptible to regression. If *β*-catenin-independent Wnt signalling is shown to contribute to progression, blockade of these events may provide a novel approach to preventing cancer development.

## Figures and Tables

**Table 1:** Sequences of primers used in RT–PCR and real-time RT–PCR analysis

	**Primers**	**TaqMan probe**
*Conventional RT–PCR*		
NKD1 (NM_033119)	F, 5′-CGCCGGGATAGAAAACTACA-3′	
	R, 5′-CTGGAGCTCTGAGACCTTGG-3′	
FZD3 (NM_017412)	F, 5′-ACATCCACCCATGCTTCTTC-3′	
	R, 5′-TCGTGACTGTTCATTGAGCC-3′	
FZD4 (NM_012193)	F, 5′-CCAACATGGCTGTTGAAATG-3′	
	R, 5′-GTCTCACTGCCTTTTCCAGG-3′	
FZD6 (NM_003506)	F, 5′-ATGAGAGAGGTGAAAGCGGA-3′	
	R, 5′-TTGGTTCTGAAGAACTGGGG-3′	
FZD10 (NM_007197)	F, 5′-CCTCCAAGACTCTGCAGTCC-3′	
	R, 5′-GACTGGGCAGGGATCTCATA-3′	
		
*Real-time PCR*		
NKD1 (NM_033119)	F, 5′-TCGCCGGGATAGAAAACTACA-3′	P, 5′-CCAATTTGGGCCTGGCTCCCC-3′
	R, 5′-CAGTTCTGACTTCTGGGCCAC-3′	
FZD3 (NM_017412)	F, 5′-CATGGAGATGTTTGGTGTTCCTT-3′	P, 5′-TCTGGGAACCTACTGCATTCCATATCTTCAGG-3′
	R, 5′-AAGTCGAGGATATGGCTCATCAC-3′	
KRT8 (NM_002273)	F, 5′-GATCGCCACCTACAGGAAGCT-3′	P, 5′-CCGGCTCTCCTCGCCCTCCA-3′
	R, 5′-ACTCATGTTCTGCATCCCAGACT-3′	
FZD6 (NM_003506)	F, 5′-ATCTGATGGGTCATTATGACCAGAGT-3′	
	R, 5′- TTTGCGAGAGGAAGAAAATGCT-3′	

**Table 2:** Summary of RT–PCR results for FZD expression in matched and unmatched adenomas (A) and normal (N) mucosa

	**Matched**			**Unmatched**		**Total (%)**		
	***N***	**A**	***P*-value**	***N***	**A**	***N***	**A**	***P*-value**
FZD1	6/8	5/8	0.5	2/5	6/11	62	58	1
FZD2	7/8	1/8	0.015	3/5	3/11	77	21	0.003
FZD3	1/8	6/8	0.03	1/5	7/11	15	68	0.005
FZD4	0/8	2/8	0.25	0/5	4/11	0	32	0.06
FZD5	7/12	6/12	0.5	ND	ND	58	50	1
FZD6	2/8	4/8	0.25	1/5	8/11	23	63	0.04
FZD7	7/12	7/12	0.6	ND	ND	58	58	1
FZD8	7/8	2/7	0.063	5/5	3/11	92	26	0.007
FZD10	1/8	2/8	0.475	0/5	5/11	8	37	0.1

ND=not performed.
